# Molecular features and tumor location interact to account for preoperative cognitive-emotional status in glioma

**DOI:** 10.3389/fneur.2026.1845406

**Published:** 2026-09-14

**Authors:** Yehong Fang, Tangsheng Ai, Ruolin Yang, Lixia Xu, Qi Shi, Zhixian Gao, Xingchao Wang, Hua Yan

**Affiliations:** 1 Huanhu Hospital Affiliated to Tianjin Medical University, Tianjin, China; 2 Department of Neurosurgery, Beijing Tiantan Hospital, Capital Medical University, Beijing, China; 3 School of Psychological and Cognitive Sciences and Mental Health, Peking University, Beijing, China; 4 Tianjin Key Laboratory of Cerebral Blood Flow Reconstruction and Head and Neck Tumor New Technology Translation, Tianjin Neurosurgical Institute, Tianjin, China; 5 The Fourth Central Hospital Affiliated to Tianjin Medical University, Tianjin, China

**Keywords:** glioma, IDH, integrated neuropathology, molecular, neuropsychology

## Abstract

**Background:**

Integrated molecular profiling has revolutionized brain tumor classification, but its ability to explain preoperative cognitive-emotional phenotypes in glioma patients relative to traditional anatomical factors remains unclear. This study investigated whether molecular features interact with tumor location to influence neuropsychological status, and whether such effects are anatomically context-dependent.

**Methods:**

We prospectively enrolled 101 newly diagnosed glioma patients and 52 matched healthy controls. All participants underwent comprehensive neuropsychological assessments. Using a hierarchical strategy, we first stratified patients by tumor location, then examined associations of molecular characteristics and clinical factors with cognitive-emotional measures within each anatomical subgroup. Finally, multivariable regression analyses with formal interaction terms were performed in the entire cohort to test molecular-location interactions.

**Results:**

No significant differences in cognitive or emotional function were observed between patients with deep insular versus superficial frontal-temporal–parietal tumors. However, within the deep insular subgroup, IDH mutation remained associated with distinct patterns of emotional and attentional function after correction for multiple comparisons. Within the superficial frontal subgroup, tumor grade and several specific molecular features showed domain-specific associations with empathy or working memory. Critically, multivariable regression confirmed significant molecular-location interactions: IDH × insular for negative affect and attentional control; grade × frontal and TERT × frontal for empathy; and 1p/19q × frontal for working memory. These findings indicate that molecular features and tumor location interact to explain neuropsychological outcomes more effectively than either factor alone.

**Conclusion:**

Molecular influences on preoperative cognitive-emotional status in glioma are anatomically context-dependent. Molecular features and tumor location interact to shape neuropsychological outcomes, supporting an integrated framework that incorporates both molecular and anatomical information into preoperative evaluation and personalized management strategies.

## 1 Introduction

Gliomas, the most common primary malignant tumors of the central nervous system, present a significant challenge in neuro-oncology not only due to their impact on survival but also because of their profound effects on patients’ neurological and neuropsychological function (1). Cognitive and emotional deficits are highly prevalent in glioma patients, arising from a complex interplay of direct tumor infiltration, neuroplastic reorganization, and potential side effects of treatments (2). These impairments severely compromise health-related quality of life, functional independence, and psychosocial wellbeing, making their understanding and management a critical component of comprehensive patient care.

Neuropsychological changes in patients with glioma are influenced by multiple factors. Tumor location has long been a focus of clinical attention. In superficial functional cortical areas, tumors may disrupt specific functions such as executive control, memory, or language. For example, frontal lobe gliomas can be associated with executive dysfunction, the temporal lobe is closely linked to memory, and parietal lobe involvement may lead to declines in visuomotor cognitive abilities (3–5). The insula, as a deep, multimodal integration area, is thought to be significantly associated not only with potential language impairments but also with interoception, emotion recognition, and social cognitive functions (6). However, such location-based associations are not unchallengeable. The slow-growing nature of these tumors allows for some degree of compensation within brain networks, and neural circuits can adapt through mechanisms of plasticity.

In recent years, the integration of molecular biomarkers has fundamentally reshaped the paradigm for glioma classification and management. The World Health Organization now defines glioma entities largely based on molecular features, including isocitrate dehydrogenase (IDH) mutation, 1p/19q co-deletion, telomerase reverse transcriptase (TERT) promoter mutation, and O6-methylguanine-DNA methyltransferase (MGMT) promoter methylation status. These markers serve as crucial prognostic and predictive factors for survival and treatment response (7). Such molecular alterations may also influence the tumor’s biological behavior, growth patterns, and interaction with the surrounding brain parenchyma, thereby potentially modulating cognitive and emotional outcomes. Nonetheless, research has predominantly focused on how molecular markers predict survival or therapeutic efficacy, while their impact on cognitive and emotional status remains poorly characterized.

Currently, several studies have suggested a general pattern that IDH-mutant gliomas are associated with more favorable cognitive performance compared to IDH-wildtype tumors (8–11). However, these studies have primarily reported global cognitive differences, whereas detailed domain-specific patterns remain to be characterized and have not reached consistent conclusions. Moreover, the cognitive implications of other molecular markers remain largely unexplored. Critically, among the limited studies that have addressed cognition, molecular markers and anatomical location have been examined as separate predictors, without formally testing whether the effect of a given molecular marker varies depending on tumor location. To address this gap, this study systematically investigated preoperative cognitive and emotional functioning in glioma patients, with a central focus on examining the associations of tumor location, clinical factors, and key molecular markers with preoperative cognitive and emotional outcomes. By demonstrating that molecular effects on neuropsychological outcomes are anatomically context-dependent, we seek to advocate for an integrated assessment framework that incorporates both molecular and anatomical information into preoperative neuropsychological evaluation and personalized patient management.

## 2 Materials and methods

### 2.1 Study design

This prospective study enrolled patients with glioma who were admitted to Beijing Tiantan Hospital between September 2019 and August 2025. All eligible patients underwent surgical treatment and were diagnosed with glioma based on histopathological confirmation. Patients were required to be treatment-naïve prior to enrollment, meaning they had not received any prior radiotherapy, chemotherapy, or surgical resection for their brain tumors. Demographically matched healthy controls (HCs) were recruited from the local community. The HCs had no history of neurological or psychiatric disorders. Detailed inclusion and exclusion criteria for all the participants, along with the recruitment flowchart, the excluded cases at each screening stage, and the timeline of neuropsychological assessments, are presented in Figure 1. A total of 101 patients with glioma and 52 healthy controls were included in the final analysis. The study protocol was reviewed and approved by the Medical Ethics Committee of Beijing Tiantan Hospital. Written informed consent was obtained from all participants prior to enrollment, in accordance with the Declaration of Helsinki.

**Figure 1:**
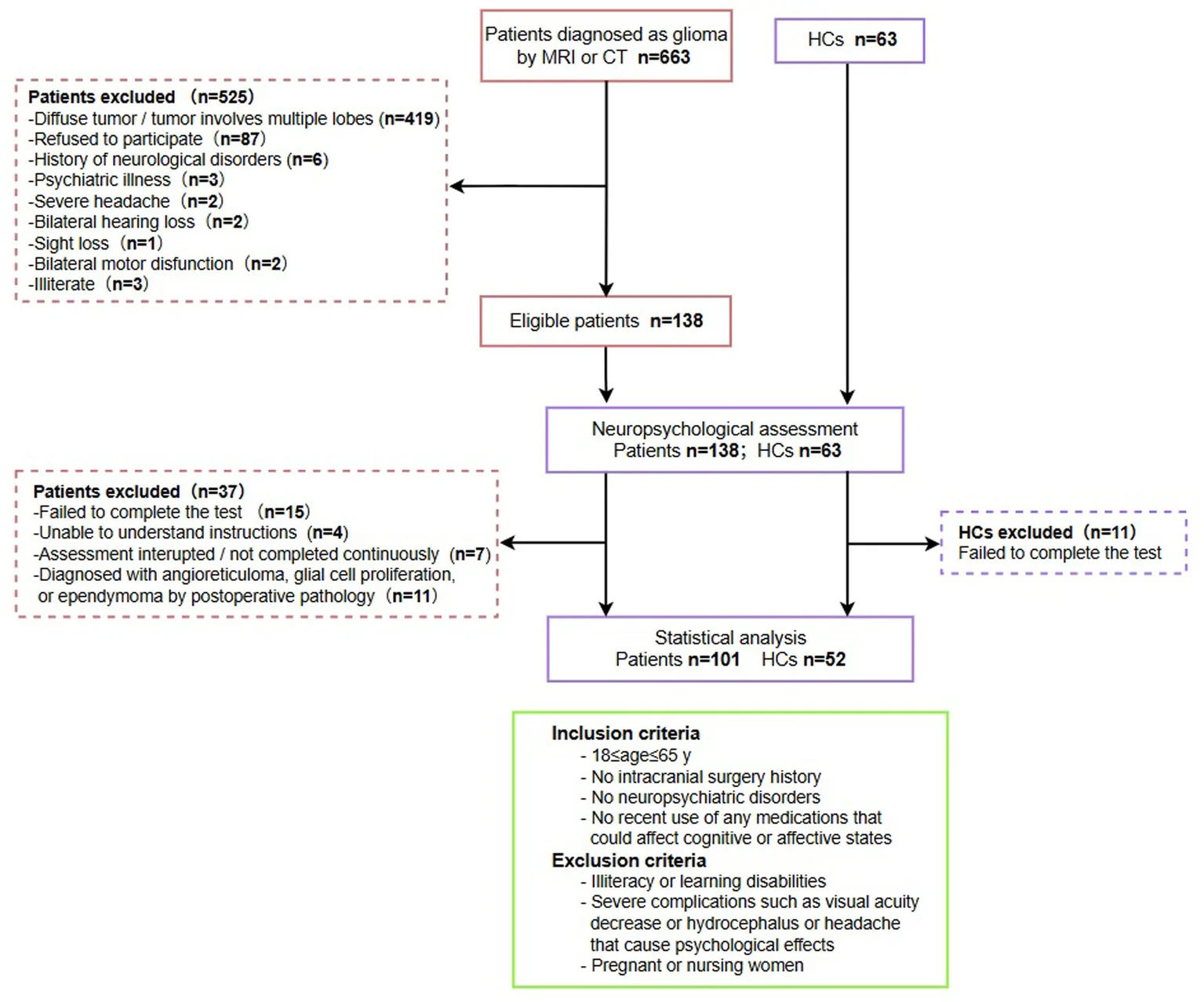
Flow diagram showing the participant enrollment and screening process, inclusion/exclusion criteria, and study design. HCs, healthy controls.

### 2.2 Clinical data collection

All patients received standardized surgical treatment and perioperative management, including standardized anesthesia protocols, postoperative care in the neurosurgical intensive care unit or ward, and early mobilization strategies. Demographic information was systematically collected for all participants, including gender, age, education level, and dominant hand as assessed by the Edinburgh Handedness Inventory (12). Comprehensive clinical data were obtained from electronic medical records and structured interviews, including medical history, smoking and alcohol consumption history, disease course defined as the interval from symptom onset to admission, initial symptoms, history of epilepsy and antiepileptic medication use, neuroimaging findings, and pathological reports.

Radiological data were acquired using a 3.0 Tesla magnetic resonance imaging scanner (TRIO; Siemens, Erlangen, Germany). The standardized imaging protocol included three-dimensional T1-weighted gradient echo sequences before and after administration of gadolinium-based contrast agent, as well as fluid-attenuated inversion recovery (FLAIR) sequences (13). All imaging parameters were optimized for glioma evaluation according to standard protocols. All surgical procedures were performed by the same experienced neurosurgeon specializing in neuro-oncology, and pathological diagnoses were confirmed by two experienced neuropathologists. Molecular pathological analyses were performed on tumor tissues obtained during surgery. IDH mutation status was detected by immunohistochemistry, 1p/19q co-deletion by fluorescence *in situ* hybridization, and TERT promoter mutation and MGMT promoter methylation by polymerase chain reaction.

### 2.3 Neuropsychological assessment

A comprehensive battery of neuropsychological scales was administered to evaluate cognitive function and emotional status in all participants. The assessment battery comprised 10 standardized instruments: the Mini-Mental State Examination (MMSE) (14) and Montreal Cognitive Assessment (MoCA) (15) for global cognitive screening; the Trail Making Test Part A(TMTA) and Part B (TMTB) (16) for attention and executive function; the Frontal Assessment Battery (FAB) (17) for frontal lobe function; the Digit Span Test (DST) from the Wechsler Adult Intelligence Scale-Fourth Edition, including forward (DST-f), backward (DST-b), and sequencing (DST-s) conditions (18) for working memory; the Attentional Control Scale (ACS) (19) for attentional control capacity; the Beck Depression Inventory (BDI) (20) for depressive symptoms; the Hamilton Anxiety Scale (HAMA) (21) for anxiety symptoms; the Positive and Negative Affect Schedule (PANAS), comprising positive (PANASp) and negative (PANASn) affect subscales (22) for affective experience; and the Interpersonal Reactivity Index (IRI) (23, 24) for empathy.

All assessments were administered by an experienced psychologist in a quiet, well-lit, and temperature-controlled room to ensure standardized testing conditions. The average completion time for the entire battery was approximately 60 min, with brief rest breaks provided as needed during the assessment to minimize fatigue effects. For patients with glioma, all neuropsychological evaluations were conducted within three days prior to surgery. Healthy controls completed the same assessment battery under identical conditions.

### 2.4 Statistical analysis

The normality of continuous variables was assessed by the Shapiro–Wilk test. For continuous variables, data conforming to a normal distribution were presented as mean ± standard deviation, whereas non-normally distributed data were expressed as median ± interquartile range. For comparisons between two independent groups, the Student’s t-test was employed when the data were approximately normally distributed and met the homogeneity of variance assumption (assessed by Levene’s test); while the Mann–Whitney U test was applied to data that did not follow a normal distribution. To compare differences between patients with glioma and healthy controls across multiple neuropsychological measures, a multivariate analysis of covariance (MANCOVA) was conducted to control for the potential confounding effects of age, gender, and education. Categorical variables were summarized as number (percentage). Group comparisons for categorical data were performed using the chi-square test, continuity-corrected chi-square test, or Fisher’s exact test, as appropriate based on expected cell frequencies. For subgroup analyses, we applied the Benjamini-Hochberg False Discovery Rate (FDR) procedure to control for Type I errors due to multiple comparisons, with a significance threshold set at FDR-adjusted *q* < 0.05. All statistical analyses were performed using SPSS software (version 22.0; IBM Corp., Armonk, NY, USA). All statistical tests were two-tailed, and the significance level was set at *p* < 0.05.

## 3 Results

### 3.1 Demographic and clinical data

A total of 101 patients with newly diagnosed glioma and 52 demographically matched healthy controls (HCs) were enrolled in this study. Detailed demographic characteristics of both groups, along with comprehensive comparisons, are presented in Supplementary Table S1. The glioma group and HCs group did not differ significantly on any demographic variables (all *p* > 0.05). However, compared with healthy controls, patients with glioma exhibited significant impairments across multiple neuropsychological domains, including global cognition, attention, executive function, working memory, as well as alterations in anxiety, positive and negative affect, and empathy. Detailed neuropsychological data for both groups are provided in Supplementary Table S2.

Among the 101 glioma patients, presenting symptoms included epilepsy, dizziness, headache, nausea or vomiting, motor or sensory deficits, language disturbances, visual impairment, and other atypical symptoms. To investigate heterogeneity within the patient population, we stratified these 101 patients based on tumor location into two subgroups: the deep location (insular) group and the superficial location (frontal-temporal–parietal) group. Detailed comparisons of demographic, clinical, pathological, and molecular characteristics between these two patient groups, along with comparisons of their neuropsychological performance, are presented in the following section. Subsequent analyses focused on patient subgroup comparisons, with healthy controls serving primarily as a reference for baseline neuropsychological performance.

### 3.2 Impact of tumor location on neuropsychological performance

To investigate whether tumor location alone influences preoperative cognitive and emotional function, we compared patients with deep location (insular) gliomas and those with superficial location (frontal-temporal–parietal) gliomas. As described in the previous section, a total of 101 patients were stratified into these two subgroups based on neuroradiological findings.

As summarized in Table 1, the two groups did not differ significantly in demographic characteristics, including age, gender, years of education, smoking, and alcohol consumption (all *p* > 0.05). Regarding clinical features, no significant differences were observed in hypertension, diabetes mellitus, operation history, history of other systemic diseases, course of disease, epilepsy, tumor laterality (dominant hemisphere involvement), or tumor size (all *p* > 0.05). In terms of pathological and molecular features, the proportion of patients with 1p/19q co-deletion was significantly higher in the deep insular group than in the superficial frontal-temporal–parietal group (53.8% vs. 26.2%, *p* = 0.017). No significant differences were found in tumor grade, Ki-67 proliferation index, or the status of other key molecular markers, including IDH mutation, MGMT promoter methylation, and TERT promoter mutation (all *p* > 0.05).

**Table 1:** Demographic, clinical, molecular characteristics and cognitive and emotional measures of glioma patients by lesion location.

Variables			Deep location (*n* = 26)	Surface location (*n* = 75)	t/Z score/χ^2^	*p*-value
Demography						
	Gender (M)		13 (50.0%)	41 (54.7%)	0.169	0.681
	Age		41.12 ± 9.31	41.19 ± 11.13	−0.029	0.977
	Education (y)		14.00 ± 4.00	14.00 ± 5.00	−0.718	0.473
	Smoking		6 (23.1%)	19 (25.3%)	0.053	0.818
	Alcohol consumption		6 (23.1%)	26 (34.7%)	1.198	0.274
Clinical feature						
	Hypertension		1 (3.8%)	11 (14.7%)	1.249	0.264
	Diabetes		1 (3.8%)	3 (4.0%)	<0.001	1.000
	Operation history		4 (15.4%)	24 (32.0%)	2.660	0.103
	History of other system disease		6 (23.1%)	34 (45.3%)	3.566	0.059
	Course of disease (mos)		2.50 ± 5.38	2.00 ± 5.00	−0.718	0.473
	Epilepsy		13 (50.0%)	32 (42.7%)	0.420	0.517
	Dominant hemisphere tumor		12 (46.2%)	40 (53.3%)	0.298	0.528
	Tumor size (cm3)		53.86 ± 70.66	37.21 ± 60.93	−1.433	0.512
Pathology						
	Grade (high)		6 (23.1%)	31 (41.3%)	2.772	0.096
	ki-67		5.00 ± 3.40	5.50 ± 20.00	−1.148	0.251
	IDH mutation		18 (69.2%)	46 (63.0%)	0.363	0.574
	1p/19q codeletion		14 (53.8%)	16 (26.2%)	5.696	*0.017*
	MGMT promoter methylation		17 (65.4%)	41 (69.5%)	0.135	0.713
	TERT promoter mutation		16 (61.5%)	29 (49.2%)	2.028	0.154
Cognitive-emotional assessment						
	Global cognitive function	MMSE	29.00 ± 1.09	29.00 ± 2.00	−0.440	0.660
		MoCA	25.19 ± 2.86	24.92 ± 2.80	0.425	0.672
	Attention	TMTA	26.50 ± 9.00	28.00 ± 15.00	−1.361	0.174
		ACS	45.54 ± 6.94	45.64 ± 6.33	−0.069	0.945
	Executive function	TMTB	65.50 ± 38.00	83.00 ± 57.00	−1.655	0.098
		FAB	15.00 ± 1.00	15.00 ± 1.00	−0.175	0.861
	Memory	DST-f	12.85 ± 2.03	12.59 ± 2.23	0.524	0.602
		DST-b	10.46 ± 2.60	10.28 ± 2.93	0.141	0.888
		DST-s	9.50 ± 1.00	9.00 ± 2.00	0.280	0.780
		DST	33.08 ± 5.08	32.35 ± 6.40	−0.444	0.657
	Anxiety	HAMA	7.54 ± 5.03	8.28 ± 5.54	0.527	0.600
	Depression	BDI	2.00 ± 5.00	3.00 ± 6.00	−0.601	0.289
	Affect	PANASp	28.35 ± 7.75	27.33 ± 7.53	0.587	0.559
		PANASn	21.08 ± 7.94	21.45 ± 7.26	−0.222	0.824
	Empathy	IRI	50.77 ± 7.11	48.77 ± 10.22	0.920	0.360

*p*-value < 0.05 was considered statistically significant. Italic type indicates statistically significant differences. ACS, Attentional Control Scale; BDI, Beck Depression Inventory; DST: Digit Span Test; DST-b, Wechsler Adult Intelligence Scale-Fourth Edition Digit Span-Backward; DST-f, Wechsler Adult Intelligence Scale-Fourth Edition Digit Span-Forward; DST-s, Wechsler Adult Intelligence Scale-Fourth Edition Digit Span-Sequencing; FAB, Frontal Assessment Battery; HAMA, Hamilton Anxiety Scale; HCs, healthy controls; IRI, Interpersonal Reactivity Index; MMSE, mini-mental state examination; MoCA, Montreal Cognitive Assessment; PANAS, Positive and Negative Affect Scale; PANASn, Positive and Negative Affect Scale-negative affect; PANASp, Positive and Negative Affect Scale-positive affect; TMTA, Trail-Making Test part A; TMTB, Trial-Making Test-part B.

Regarding cognitive and emotional performance, patients with tumors located in the deep insular region and those with tumors in the superficial frontal-temporal–parietal regions showed comparable scores across all assessed domains, including global cognition (MMSE, MoCA), attention (TMTA, ACS), executive function (TMTB, FAB), memory (DST subtests and total score), depression (BDI), anxiety (HAMA), positive and negative affect (PANASp, PANASn), and empathy (IRI). No significant differences were observed between the two groups in any of these measures (all *p* > 0.05).

Given the significant between-group difference in the proportion of 1p/19q co-deletion, a molecular feature potentially associated with neuropsychological function, we performed a sensitivity analysis using analysis of covariance (ANCOVA) with 1p/19q status as a covariate to evaluate whether this imbalance confounded the comparison of neuropsychological performance by tumor location. As shown in Table 2, after adjusting for 1p/19q status, the main effect of tumor location remained non-significant for all neuropsychological measures (all *p* > 0.05), confirming the robustness of the finding that anatomical location alone does not substantially influence preoperative neuropsychological function. Notably, 1p/19q status itself exhibited significant independent effects on several cognitive domains, primarily in memory (DST-f, DST-b, DST; all *p* < 0.05), and reached borderline significance in executive function (TMTB; *p* = 0.05). This finding suggests that molecular characteristics may be distinctly associated with specific dimensions of neuropsychological function. These results provide a rationale for further exploring cognitive and emotional heterogeneity within molecular subgroups stratified by anatomical location, which will be detailed in the following sections.

**Table 2:** Effects of tumor location and 1p/19q status on neuropsychological performance (ANCOVA).

Cognitive-emotional assessment		Location		1p/19q status	
Global cognitive function		F score	*p*-value	F score	*p*-value
	MMSE	0.214	0.645	0.001	0.975
	MoCA	0.031	0.860	0.987	0.323
Attention					
	TMTA	1.318	0.254	3.575	0.062
	ACS	0.003	0.959	0.245	0.622
Executive function					
	TMTB	1.118	0.293	3.930	0.050
	FAB	<0.001	1.000	0.030	0.862
Memory					
	DST-f	0.031	0.862	8.758	*0.004*
	DST-b	0.313	0.577	12.734	*0.001*
	DST-s	0.154	0.695	1.099	0.297
	DST	0.039	0.843	9.476	*0.003*
Anxiety					
	HAMA	0.281	0.597	0.046	0.831
Depression					
	BDI	0.459	0.500	0.474	0.493
Affect					
	PANASp	0.305	0.582	0.004	0.952
	PANASn	0.021	0.884	2.349	0.129
Empathy					
	IRI	1.006	0.318	0.224	0.637

Data were analyzed using ANCOVA with 1p/19q status as covariate. Significant *p*-values (< 0.05) are indicated in italics. ACS, Attentional Control Scale; BDI, Beck Depression Inventory; DST: Digit Span Test; DST-b, Wechsler Adult Intelligence Scale-Fourth Edition Digit Span-Backward; DST-f, Wechsler Adult Intelligence Scale-Fourth Edition Digit Span-Forward; DST-s, Wechsler Adult Intelligence Scale-Fourth Edition Digit Span-Sequencing; FAB, Frontal Assessment Battery; HAMA, Hamilton Anxiety Scale; HCs, healthy controls; IRI, Interpersonal Reactivity Index; MMSE, mini-mental state examination; MoCA, Montreal Cognitive Assessment; PANAS, Positive and Negative Affect Scale; PANASn, Positive and Negative Affect Scale-negative affect; PANASp, Positive and Negative Affect Scale-positive affect; TMTA, Trail-Making Test part A; TMTB, Trial-Making Test-part B.

### 3.3 Neuropsychological differences based on clinical and molecular characteristics in the deep insular subgroup

Within the deep insular subgroup, we further investigated whether heterogeneity in clinical features and molecular characteristics was associated with distinct cognitive and emotional phenotypes. Patients were stratified according to various clinical variables (dominant hemisphere involvement, epilepsy status) and key molecular markers (IDH mutation, 1p/19q co-deletion, MGMT promoter methylation, TERT promoter mutation, tumor grade). For each comparison, we first confirmed that the compared subgroups were well-balanced on demographic characteristics (age, gender, years of education, smoking, and alcohol consumption) and other potential confounders, as illustrated in Figure 2 and detailed in Supplementary Tables S3, S4. Given that no significant imbalances were observed in these baseline factors, independent t-tests or Mann–Whitney U tests were used to compare neuropsychological performance between subgroups, with subsequent FDR adjustment for multiple comparisons. The results are summarized in Table 3 and described below.

**Figure 2:**
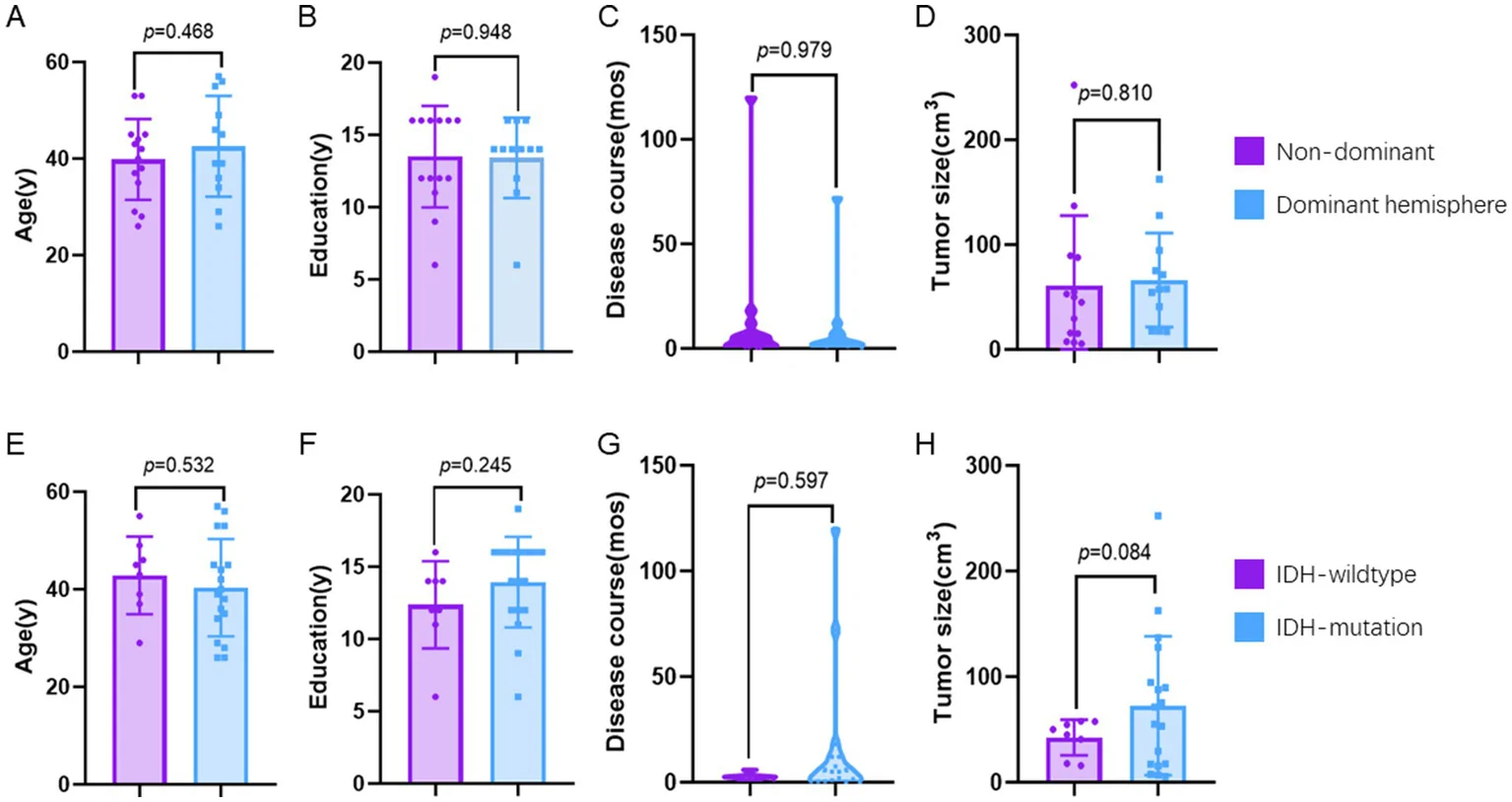
Comparison of baseline demographic and clinical characteristics of patients within the deep insular subgroup. Patients were stratified by dominant hemisphere **(A–D)** and IDH mutation status **(E–H)**.

**Table 3:** Comparisons of neuropsychological test scores by clinical and molecular features in the insular lobe subgroup (*n* = 26).

Influencing factor (Group, *n*)	Domain and test showing significant difference	t/Z score	*p*-value	*q*-value
Laterality	Global cognition-MoCA ^1^	3.815	0.001	0.008
Dominant hemisphere (*n* = 12)				
Non-dominant hemisphere (*n* = 14)				
IDH mutation status	Attention-ACS ^2^	6.647	<0.001	0.008
Mutant (*n* = 18)	Affect-PANASp ^3^	−3.790	*0.001*	0.008
Wild-type (*n* = 8)	Affect-PANASn ^2^	4.265	*<0.001*	0.008
	Empathy-IRI ^2^	2.402	*0.024*	0.144

Only comparisons that yielded statistically significant differences are shown in this table. Significant p-values (< 0.05) and FDR-adjusted *q*-values (< 0.05) are indicated in italics. The two groups showed no significant differences in demographic factors. ^1^ Dominant hemisphere group scored higher than non-dominant hemisphere group. ^2^ Wild-type group scored higher than IDH-mutant group. ^3^ Wild-type group scored lower than IDH-mutant group.

Patients with tumors involving the dominant hemisphere exhibited significantly lower scores on global cognitive assessment (MoCA, *p* = 0.001, FDR-adjusted *q* = 0.008) compared to those with non-dominant hemisphere involvement. No significant differences were observed in other cognitive or emotional domains between these two groups.

Additionally, compared to patients with IDH-wildtype tumors, those with IDH-mutant tumors showed significantly higher positive affect (PANASp, p = 0.001, FDR-adjusted *q* = 0.008) and lower negative affect (PANASn, *p* < 0.001, FDR-adjusted *q* = 0.008). However, the IDH-mutant group performed worse on attention (ACS, *p* < 0.001, FDR-adjusted *q* = 0.008). A difference in empathy (IRI, *p* = 0.024) was also observed between the two groups, but it did not remain significant after FDR correction (FDR-adjusted *q* = 0.144).

When patients were grouped according to epilepsy status, tumor grade, 1p/19q co-deletion status, MGMT promoter methylation status, or TERT promoter mutation status, no significant differences were observed in any neuropsychological scales (all *p* > 0.05).

### 3.4 Neuropsychological differences based on clinical and molecular characteristics in the superficial frontal subgroup

We further focused on the frontal subgroup, a representative superficial tumor location, to analyze the potential impact of clinical and molecular factors on cognitive and emotional performance within this anatomically defined cohort. Following the same analytical approach as in the insular subgroup, we first confirmed that compared subgroups were well-balanced on demographic characteristics (age, gender, years of education, smoking, and alcohol consumption) and other potential confounders for each stratification variable. Baseline comparisons are presented in Figure 3 and detailed in Supplementary Tables S5–S9. After establishing comparability between groups, independent t-tests or Mann–Whitney U tests were used to compare neuropsychological performance, with subsequent FDR adjustment for multiple comparisons. The main findings are summarized in Table 4 and described below.

**Figure 3:**
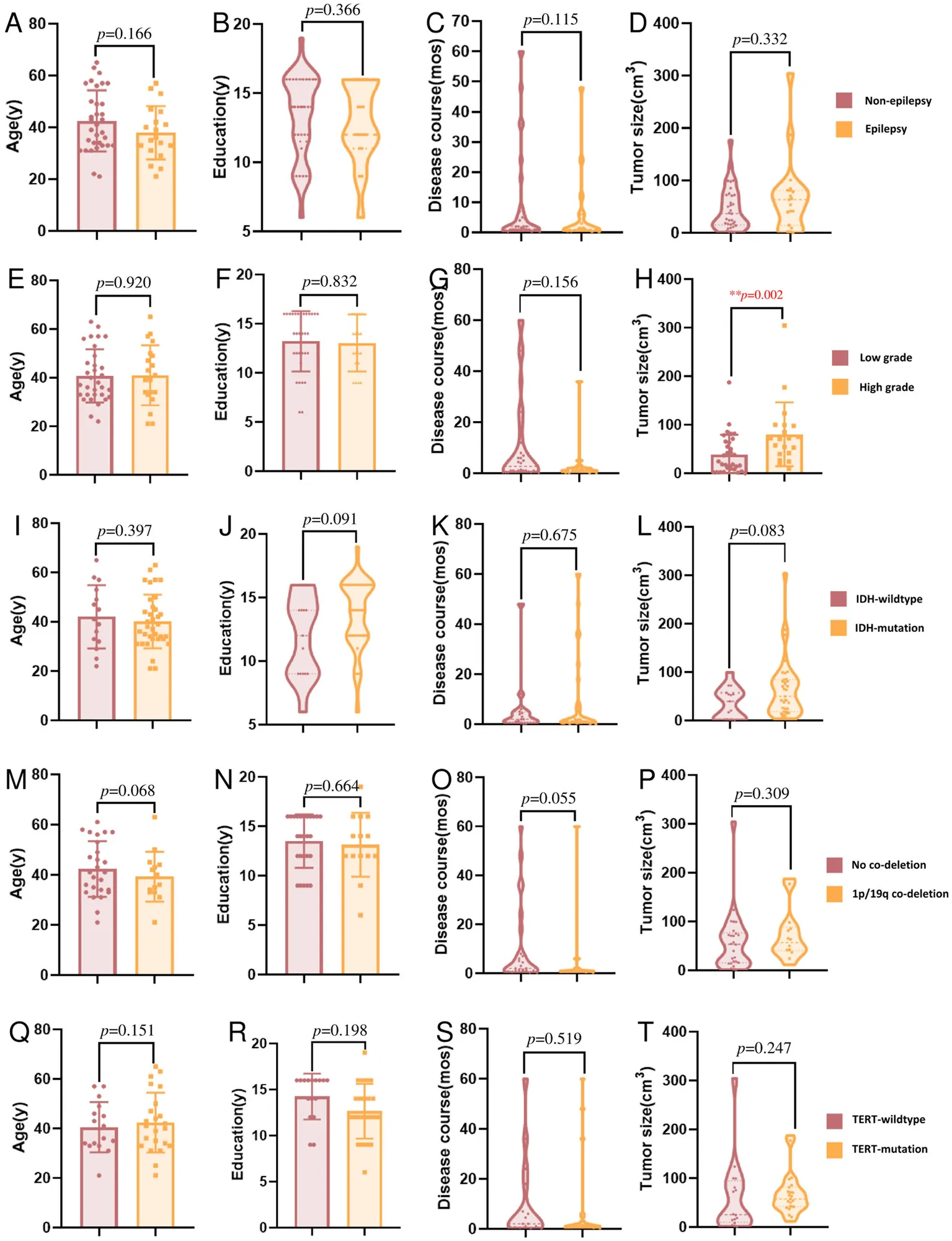
Comparison of baseline demographic and clinical characteristics of patients within the superficial Frontal subgroup. Patients were stratified by epilepsy **(A–D)**, tumor grade **(E–H)**, IDH mutation (I–L), 1p/19q co-deletion **(M–P)**, and TERT mutation status **(Q–T)**.

**Table 4:** Comparisons of neuropsychological test scores by clinical and molecular features in the frontal lobe subgroup (*n* = 52).

Influencing factor (Group, n)	Domain and test showing significant difference	t/Z score	*p*-value	*q*-value
Epilepsy				
Yes (*n* = 19)	Global cognition-MoCA ^1^	2.434	*0.021*	0.158
	Memory-DST-s ^1^	2.761	*0.008*	0.083
No (*n* = 33)	Executive function-FAB ^1^	2.270	*0.028*	0.191
	Affection-PANASp ^1^	2.700	*0.010*	0.083
Grade				
High (*n* = 20)	Empathy-IRI ^2^	4.069	*<0.001*	*0.015*
Low (*n* = 32)				
1p/19q Co-deletion				
Codeleted (*n* = 19)	Attention-TMTA ^3^	2.659	*0.010*	0.083
	Memory-DST-f ^4^	−3.494	*0.001*	*0.015*
Intact (*n* = 33)	Memory-DST-b ^4^	−3.574	*0.001*	*0.015*
	Memory-DST ^4^	−3.592	*0.001*	*0.015*
TERT Promoter Mutation				
Mutant (*n* = 27)	Empathy-IRI ^5^	4.248	*<0.001*	*0.015*
Wild-type (*n* = 25)				
IDH Mutation Status				
Mutant (*n* = 36)	Attention-TMTA ^6^	2.852	*0.006*	0.075
Wild-type (*n* = 16)				

Only comparisons that yielded statistically significant differences are shown in this table. Significant *p*-values (< 0.05) and FDR-adjusted *q*-values (< 0.05) are indicated in italics. The two groups showed no significant differences in demographic factors. Low-grade: WHO grade 1–2; High-grade: WHO grade 3–4. ^1^ No-epilepsy group scored higher than epilepsy group. ^2^ Low-grade group scored higher than high-grade group. ^3^ Co-deletion group required less time to complete the task. ^4^ Co-deletion group scored higher than non-codeletion group. ^5^ Wild-type group scored higher than TERT-mutant group. ^6^ IDH-mutant group required less time to complete the task.

Regarding epilepsy status, patients without epilepsy exhibited better performance in global cognition (MoCA, *p* = 0.021), memory (DST-s, *p* = 0.008), executive function (FAB, *p* = 0.028), and positive affect (PANASp, *p* = 0.010) compared with those with epilepsy. However, none of these differences survived FDR adjustment (all FDR-adjusted *q* > 0.05). With respect to tumor grade, patients with low-grade tumors demonstrated significantly higher levels of empathy (IRI, *p* < 0.001, FDR-adjusted *q* = 0.0015) than those with high-grade tumors.

In terms of molecular characteristics, several associations were observed. Patients with 1p/19q co-deletion showed better performance in memory (DST-f, *p* = 0.001, FDR-adjusted *q* = 0.0015; DST-b, *p* = 0.001, FDR-adjusted *q* = 0.0015; DST, *p* = 0.001, FDR-adjusted *q* = 0.0015) compared with those without co-deletion. A difference in attention (TMTA, *p* = 0.010) was also observed between the two groups, but it did not withstand FDR adjustment (FDR-adjusted *q* = 0.083). Patients with TERT-wildtype tumors exhibited higher empathy scores (IRI, *p* < 0.001, FDR-adjusted *q* = 0.0015) than those with TERT-mutant tumors. Additionally, patients with IDH-mutant tumors completed the attention task in less time (TMTA, *p* = 0.006) compared with IDH-wildtype patients, but it was non-significant after FDR adjustment (FDR-adjusted *q* = 0.075).

No significant differences in neuropsychological performance were observed for tumor laterality (dominant vs. non-dominant hemisphere involvement) or MGMT promoter methylation status (all *p* > 0.05).

### 3.5 Interactive effects of molecular features and tumor location on neuropsychological outcomes

To circumvent the analytical limitations of fragmented subgroup analyses, we constructed hierarchical multiple regression models in the full patient cohort based on FDR-corrected significant scale indicators, directly testing the effects of molecular pathology and tumor location on cognitive and emotional outcomes. Each model controlled for demographic variables and included main effects of clinical and molecular factors, as well as their interaction terms. Results are presented in Table 5.

**Table 5:** Hierarchical multivariable regression models examining predictors of key neuropsychological outcomes.

Predictor		PANASp	PANASn	ACS	IRI	DST-f	DST-b	DST	IRI
Block 1: Covariates									
	Age (B)	0.084	−0.104	−0.069	0.060	−0.027	−0.052*	−0.113*	0.104
	Gender (B)	0.679	1.576	1.041	1.562	0.621	0.944	1.881	1.548
	Education (B)	−0.087	0.371	−0.466*	0.099	0.183*	0.206*	0.548*	−0.085
Block 2: Main effects									
	IDH (B)	0.582	−1.199	−0.244					
	Grade (B)				−5.311*				
	1p/19q codeletion (B)					0.180	0.900	0.325	
	TERT (B)								1.090
	Insular (B)	−5.502	7.070*	7.137*					
	Frontal (B)				−2.775	−0.710	−0.343	−2.059	3.533
	Temporal (B)	0.454	0.458	−2.405	0.882	0.189	0.523	0.282	0.998
	Parietal (B)	1.786	−1.990	−1.496	−1.422	−1.541	−1.437	−3.811	−0.797
Block 3: Interactions									
	IDH × Insular (B)	9.714*	−11.015*	−11.248*					
	Grade × Frontal (B)				−11.000*				
	1p/19q × Frontal (B)					1.780	1.582	5.123*	
	TERT × Frontal (B)								−12.206*
Model summary									
	R^2^ (Block 1)	0.017	0.036	0.067	0.023	0.137	0.165	0.190	0.023
	δR^2^ (Block 2)	0.040	0.066	0.068	0.111*	0.092*	0.100*	0.081*	0.108*
	δR^2^ (Block 3)	0.068*	0.091*	0.124*	0.070*	0.031	0.015	0.033*	0.089*
	Overall *p*	0.126	0.009*	<0.001*	0.006*	<0.001*	<0.001*	<0.001*	0.003*

B values are unstandardized coefficients from the final model (Block 3). *p* < 0.05 was considered statistically significant and is indicated in italics with an *. Demographic variables, including age, gender, and education, were entered as covariates in Block 1. Main effects of molecular markers, clinical factors, and location indicators were entered in Block 2. Interaction terms were entered in Block 3. All predictors were mean-centered prior to creating interaction terms to reduce multicollinearity; all variance inflation factors (VIF) were < 4, indicating no significant multicollinearity.

For insular tumors, when PANASn served as the dependent variable, the model was significant (overall *p* = 0.009), with a significant interaction term (IDH × Insula, *p* = 0.002) and a significant main effect of insular location (*p* = 0.017). When ACS served as the dependent variable, the model was significant (overall *p* < 0.001), with a significant interaction term (IDH × Insula, *p* < 0.001), alongside significant main effects of education years (*p* = 0.022) and insular location (*p* = 0.004). These findings suggest that the interaction between IDH mutation status and insular location jointly drives the effects on negative affect and attention. Additionally, when PANASp was the dependent variable, the interaction term was also observed to be significant (*p* = 0.009), but the overall model showed weak explanatory power (overall *p* = 0.126).

For frontal tumors, when IRI was the dependent variable, both the interaction terms for Grade × Frontal (*p* = 0.006) and TERT × Frontal (*p* = 0.002) were significant, with valid models (overall *p* = 0.006; overall *p* = 0.003), indicating that tumor grade and TERT mutation exert location-specific effects on empathy within the frontal lobe. When DST was the dependent variable, the interaction term for 1p/19q codeletion × Frontal and the model were both significant (*p* = 0.039, overall *p* < 0.001). However, its subtests did not show consistently significant results and were substantially influenced by demographic factors such as age and education years (DST-f Block1 R^2^ = 0.137; DST-b Block1 R^2^ = 0.165).

## 4 Discussion

In this study, we enrolled 101 patients with newly diagnosed glioma and 52 healthy controls, with a primary focus on comparing the neuropsychological profiles of patients harboring tumors in the deep insular region versus those with tumors in the superficial frontal-temporal–parietal regions. The results revealed no significant differences between the two groups in either global or domain-specific cognitive and emotional scores. However, within each anatomical subgroup, both molecular characteristics and specific clinical factors were significantly associated with cognitive and emotional functions. Multivariate analysis further demonstrated that these factors interact with anatomical location to collectively shape the preoperative neuropsychological phenotype in glioma patients. These observations underscore the need to integrate information on tumor location, molecular features, and clinical factors in the preoperative neuropsychological evaluation of these patients.

### 4.1 Cognitive and emotional function in glioma patients: from location determinism to multi-factorial convergence

In the present study, one of the most critical findings is the absence of significant differences in preoperative cognitive and emotional function between patients with deep insular tumors and those with superficial frontal-temporal–parietal tumors either globally or within any specific domain. This result diverges from predictions based on classical “location-function” models derived from acute focal brain injuries such as stroke (25), which posit that damage to a specific brain region leads to correspondingly specific functional deficits.

Gliomas are slow-growing, infiltrative tumors. Owing to their chronic nature, they allow for functional reorganization and compensation through neural plasticity within brain networks (26). Consequently, the macroscopic location of a tumor may not directly “disrupt” function but rather establish an “anatomical context of influence.” Within this context, the cognitive and emotional phenotype is largely shaped by the tumor’s intrinsic biological characteristics and the clinical states it induces, such as molecular features and epilepsy.

Notably, the distribution of these molecular features within the brain is not random; studies have confirmed that distinct molecular subtypes of glioma exhibit specific anatomical predilections (27). In previous studies investigating the role of location, if these more fundamental molecular and clinical variables were not adequately balanced, any observed “location effect” might have been confounded by the aggregation of specific molecular subtypes within particular brain regions. Admittedly, our anatomical categorization is relatively coarse and may dilute subtle location-specific effects. Our supplementary four-subgroup analysis (Supplementary Table S10) still revealed no significant differences, albeit with reduced statistical power due to smaller sample sizes. By rigorously matching baseline characteristics and conducting subgroup analyses, the present study sought to disentangle these confounding factors. This approach suggests that the independent contribution of macroscopic location per se may be limited, and highlights the significant role of molecular and clinical factors when the anatomical context is taken into account.

### 4.2 The unignorable role of molecular features: from prognostic markers to phenotype shapers

Although the significance of molecular markers in the diagnosis and prognosis of glioma has been extensively studied, their specific impact on patients’ preoperative cognitive and emotional functions, particularly within distinct neuropsychological domains such as attention, working memory, and empathy, remains insufficiently elucidated. Here, our subgroup analyses provide novel clues suggesting that key molecular alterations, including IDH mutation, 1p/19q co-deletion, and TERT promoter mutation, not only define the biological essence of the tumor but may also directly participate in shaping heterogeneous neuropsychological phenotypes by interfering with specific brain networks.

Our findings reveal a pronounced anatomical context-dependency in the effects of IDH mutation. In the superficial frontal subgroup, patients with IDH-mutant tumors demonstrated better attentional control, aligning with the classical view that this mutation is associated with favorable prognosis and cognitive resilience (28, 29). However, within the deep insular subgroup, we observed a more complex, “dissociated” pattern: while IDH-mutant patients reported more positive emotional experiences (higher positive affect and lower negative affect), they performed worse on tasks measuring attentional control and empathy. We speculate that this seemingly paradoxical phenotype may stem from the unique role of the insula as a hub for socio-emotional-cognitive integration (30). In this specific locale, IDH mutation might induce excessive structural compensation (31). This compensatory attempt to maintain basic functions could come at the cost of advanced network integration efficiency. Concurrently, the inherent vulnerability of the insular region to damage within the salience network (32) might, when combined with the above, specifically disrupt the higher-order network integration responsible for complex attention and social cognition. In contrast, the more primitive limbic circuits underlying basic emotional processing may be less affected by this decline in network efficiency (33), potentially even showing relative preservation or a positive bias due to IDH-related neuromodulatory changes. This leads to the observed dissociation phenomenon within the insular subgroup: a decline in higher-order cognition alongside preserved or even improved basic emotional processing. It should be noted that, given the associations involving empathy in the insular subgroup and attention in the frontal subgroup did not survive FDR correction, these exploratory trends also warrant validation in larger, independent cohorts.

Furthermore, other molecular markers also demonstrated differential impacts on specific cognitive domains. Within the frontal subgroup, the absence of 1p/19q co-deletion was associated with poorer performance in both attention and working memory. Given that 1p/19q co-deletion is typically linked to a more indolent tumor biology characterized by slower growth and less invasive behavior (34), we hypothesize that tumors lacking this co-deletion possess greater inherent invasiveness. This heightened aggressivity may consequently lead to earlier and more extensive structural disruption of the frontoparietal network, which is critical for sustaining attention and executive functions, as well as the core circuitry involving multiple brain regions that underlies working memory (35, 36). However, the relationship between 1p/19q co-deletion and attention did not reach statistical significance after FDR adjustment. Future studies with more focused attention assessments could help clarify this potential link.

Moreover, the presence of a TERT promoter mutation was correlated with diminished empathic capacity. TERT mutation is a key driver event that confers sustained proliferative potential and invasive tumor growth (37, 38). Empathy, as a higher-order social cognitive function, relies on the fine-grained integration of a distributed large-scale network encompassing brain regions such as the prefrontal and temporal cortices, anterior cingulate gyrus, and insula (39, 40). Therefore, the highly invasive growth pattern signaled by TERT mutation may pose a particular threat to the integrity of these widespread and vulnerable long-range functional connections. This could preferentially compromise advanced functions such as empathy, which demand sophisticated collaboration across complex neural networks.

In summary, our findings suggest that distinct molecular features may predispose tumors to specific patterns of invasion, ranging from focused local disruption to widespread network erosion, thereby selectively impairing cognitive and emotional domains that depend on the corresponding neuroanatomical substrates. This work deepens our understanding of the links between glioma molecular biology and neuropsychological outcomes and underscores the necessity of incorporating molecular information into comprehensive clinical assessments.

### 4.3 Identification of key clinical factors and their implications

In the preceding sections, we have discussed the role of molecular features in shaping the neuropsychological phenotypes of glioma patients. In addition to these molecular characteristics, we also identified several clinical factors that exert substantial influences on cognitive and emotional function, including dominant hemisphere involvement, epilepsy, and tumor grade. These factors are often more readily identifiable at the time of diagnosis and may serve as important indicators for early risk stratification and clinical management. In this section, we discuss these key clinical factors in detail, focusing on their underlying mechanisms and clinical implications.

Within the insular subgroup, patients with tumors located in the dominant hemisphere exhibited significantly lower global cognitive function. The insula serves as an integration hub for maintaining advanced cognitive functions such as attention, memory, and executive control (6, 41, 42). Particularly within the dominant hemisphere, it exhibits dense connections with the language network and is deeply involved in high-level cognitive activities including language processing and logical reasoning (43). When a tumor invades the insula of the dominant hemisphere, its disruptive effects are amplified and propagate through functional connections to the broader language and cognitive control networks. This cascade of disruption leads to a comprehensive decline in the efficiency of information integration and cognitive resource allocation, ultimately manifesting as a reduction in overall cognitive function.

In addition to the impact of dominant hemisphere involvement, epilepsy status also emerged as a significant factor influencing neuropsychological performance. Within the frontal subgroup, patients without epilepsy exhibited superior performance in global cognition, memory, executive function, and positive affect. This finding aligns closely with previous research indicating that epileptic seizures and abnormal neuronal discharges disrupt the normal electrophysiological activity of frontal networks (44), thereby continuously weakening the neural synchronization and stability essential for advanced cognitive processes such as memory and executive control (45, 46). Our study further reveals that this negative impact is extensive, concurrently affecting both cognitive domains and the positive dimension of emotion. Therefore, epilepsy is not merely a clinical symptom requiring management but also serves as a key clinical marker for predicting broad neuropsychological risks (47, 48). However, it should be pointed out that the above differences did not retain statistical significance after FDR correction, therefore these findings are regarded as preliminary and exploratory in nature. Given that they are broadly consistent with the established view that epilepsy may predispose patients to a less favorable neuropsychological profile, we consider that future studies with larger sample sizes are needed to determine whether this association reflects a genuine but modest effect that our current analysis was underpowered to detect.

Furthermore, tumor grade was also found to be associated with specific cognitive outcomes. Within the frontal subgroup, patients with low-grade tumors demonstrated higher empathic capacity. Tumor grade serves as a direct indicator of biological aggressiveness: low-grade gliomas exhibit slower growth, which allows a time window for neural remodeling and functional compensation (49). This slower progression may better preserve the integrity of the distributed networks underlying social cognition (50). In contrast, the rapid and destructive growth pattern of high-grade tumors is more likely to erode these delicate and vulnerable long-range connections, thereby impairing advanced social functions such as empathy, which rely on complex network collaboration (51, 52).

Taken together, these findings support that in the preoperative neuropsychological assessment and counseling of glioma patients, molecular markers such as IDH mutation, 1p/19q codeletion, and TERT promoter mutation, along with clinical factors including dominant hemisphere involvement, epilepsy, and tumor grade, should be given at least equal, if not greater, consideration than tumor location. Overlooking these factors or their interplay may lead to underestimation or misjudgment of the actual neuropsychological risk. For patients with distinct molecular or clinical profiles or complications, cognitive rehabilitation strategies and symptom management plans may require individualized consideration tailored to their specific risk factors.

### 4.4 Clinical implications and translational values

The findings of this study carry several implications for clinical practice. First, our findings suggest that the conventional emphasis on tumor location as a primary predictor of cognitive deficits in glioma patients may need to be reconsidered. While anatomical location undoubtedly provides valuable context, our results indicate that molecular characteristics and key clinical factors, along with their interaction with location, may exert more substantial influences on neuropsychological function. This highlights the need for a more integrated preoperative assessment framework that combines anatomical, molecular, and clinical information.

Second, the identification of location-specific cognitive–emotional profiles associated with certain molecular alterations offers the potential for more precise patient counseling. For instance, patients with IDH-mutant tumors located in the insula may benefit from preoperative education regarding possible declines in attention and empathy, alongside reassurance about relatively preserved emotional well-being. Similarly, the finding that TERT promoter mutation in frontal tumors is associated with reduced empathic capacity may help clinicians anticipate social cognitive difficulties that could impact family dynamics and postoperative recovery.

Third, the associations observed with clinical factors and neuropsychological function underscore their value as readily available indicators for early risk stratification. In particular, the interactive effect between tumor grade and frontal location on empathy suggests that grade information not only serves as a prognostic marker, but may also assist neurosurgeons in planning surgical strategies with greater caution regarding the preservation of cortical and subcortical functional areas.

Finally, these findings support a multidisciplinary approach to preoperative neuropsychological evaluation, involving collaboration among neurosurgeons, neuropsychologists, and neuropathologists. By integrating molecular pathology reports into cognitive assessment protocols, clinicians can develop individualized rehabilitation strategies and symptom management plans that address the specific vulnerabilities associated with each patient’s tumor biology.

### 4.5 Study considerations and future perspectives

Several strengths of this study should be acknowledged. To the best of our knowledge, this is the first study to systematically integrate anatomical location, molecular characteristics, and key clinical factors in the analysis of preoperative neuropsychological function in glioma patients. Unlike previous studies that have primarily focused on either tumor location alone or molecular features alone, our approach first stratified patients by tumor location and then further examined cognitive and emotional differences within each location based on molecular and clinical subgroups. Beyond these subgroup comparisons, we further performed multivariate regression analyses, especially with the inclusion of interaction terms, allowing us to estimate the independent and interactive contributions of these factors to neuropsychological outcomes. This hierarchical analytical framework enabled us to disentangle the relative contributions of these factors and to reveal the synergistic interplay between molecular features and anatomical location in shaping neuropsychological outcomes, representing a methodological innovation that has not been previously applied in this context.

In addition to this conceptual advancement, several methodological strengths further enhance the validity of our findings. The prospective design, combined with a relatively large sample size and a comprehensive neuropsychological battery covering multiple cognitive and emotional domains, allowed for detailed subgroup analyses. The inclusion of demographically matched healthy controls provided a robust reference for characterizing the overall cognitive profile of glioma patients.

Several limitations also warrant consideration. First, although the overall sample size was adequate, some subgroup analyses involved relatively small numbers, which may have limited statistical power to detect certain subtle effects and may have led to unstable effect estimates. Second, as all participants were recruited from a single center, the generalizability of our conclusions to other populations may be limited, and data on peritumoral edema, corticosteroid use, and antiseizure medication types were not available, which may confound the observed associations. Future multicenter studies with more diverse patient populations and more comprehensive clinical data collection are needed to validate and extend our findings. Third, the neuropsychological assessment relied on scale-based measures; future studies incorporating multimodal neuroimaging data would be valuable to directly examine the underlying neural networks implicated in our findings. Fourth, the molecular markers were examined using routine clinical assays that yield binary classifications, while future work employing more comprehensive techniques will be needed to identify specific mutation variants and methylation patterns for more refined analyses and clinical translation. Despite these limitations, our results provide preliminary but valuable insights into the preoperative neuropsychological profiles of glioma patients and highlight the important roles of molecular markers and key clinical factors, as well as their interaction with location, and the subgroup-specific observations await validation in larger, independent cohorts.

## Data Availability

The original contributions presented in the study are included in the article/Supplementary material, further inquiries can be directed to the corresponding author/s.
