# Supplementary material for: Molecular features and tumor location interact to account for preoperative cognitive-emotional status in glioma
Source: Front Neurol. 2026 Sep 14;17:1845406. doi: 10.3389/fneur.2026.1845406 (PMC13616668; doi:10.3389/fneur.2026.1845406)
Supplement: Supplementary file 1 [file Table_1.docx]

Supplementary Tables

**Table S1. Demographic and clinical characteristics of the participants**

| **Variable** |  |  | **Patients(n=101)** | **HCs(n=52)** | ***p*-value** |
| --- | --- | --- | --- | --- | --- |
| Gender(M) | |  | 54(53.5%) | 23(44.2%) | 0.279 |
| Age |  |  | 41.17±10.64 | 38.54±11.52 | 0.161 |
| Education(y) | |  | 14.00±5.00 | 12.50±7.00 | 0.217 |
| Dominant hand(R) | |  | 90(89.1%) | 49(94.2%) | 0.456 |
| Hypertension | |  | 12(11.9%) | 3(5.8%) | 0.229 |
| Diabetes |  |  | 4(4.0%) | 1(1.9%) | 0.848 |
| Operation history | |  | 28(27.7%) | 9(17.3%) | 0.154 |
| History of other system disease | | | 40(39.6%) | 15(28.8%) | 0.189 |
| Smoking |  |  | 25(24.8%) | 7(13.5%) | 0.104 |
| Alcohol comsumption | | | 32(31.7%) | 10(19.2%) | 0.102 |
| Disease course(mos) | |  | 2.00±5.00 | - | - |
| Initial Symptom | |  |  |  |  |
|  | Asymptomatic | | 18(17.8%) | - | - |
|  | Epilepsy |  | 37(36.6%) | - | - |
|  | Dizziness |  | 12(11.9%) | - | - |
|  | Headache |  | 16(15.8%) | - | - |
|  | Nausea/vomit | | 2(2.0%) | - | - |
|  | Motor/sensory dysfunction | | 9(8.9%) | - | - |
|  | Language disorder | | 4(4.0%) | - | - |
|  | Hypopsia |  | 1(1.0%) | - | - |
|  | Other symptoms | | 2(2.0%) | - | - |
| Epilepsy |  |  | 45(44.6%) | - | - |
| AEDs |  |  | 37(36.6%) | - | - |
| Laterality(R) | |  | 48(47.5%) | - | - |
| Dominant hemisphere tumor | | | 52(51.5%) | - | - |
| Tumor size(cm3) | |  | 40.97±60.53 | - | - |
| Location |  |  |  |  |  |
|  | Frontal lobe | | 52(51.5%) | - | - |
|  | Temporal lobe | | 17(16.8%) | - | - |
|  | Parietal lobe | | 6(5.9%) | - | - |
|  | Occipital lobe | | 0 | - | - |
|  | Insular lobe | | 26(25.7%) | - | - |
| Tumor grade | |  |  |  |  |
|  | WHO 1 |  | 11(10.9%) | - | - |
|  | WHO 2 |  | 53(52.5%) | - | - |
|  | WHO 3 |  | 25(24.8%) | - | - |
|  | WHO 4 |  | 12(11.9%) | - | - |

AEDs, antiepileptic drugs; F, female; HCs, healthy controls; L, left; M, male; R, right.

**Table S2. Comparison of Cognitive and Emotional Measures Between Glioma Patients and Healthy Controls (MANCOVA)**

| **Variables** |  | **Patients(n=101)** | **HCs(n=52)** | **F score** | ***p*-value** | **partial η2** |
| --- | --- | --- | --- | --- | --- | --- |
| General Global cognitive Function | | |  |  |  |  |
|  | MMSE | 28.52±1.62 | 29.00±1.12 | 5.11 | *0.027* | 0.034 |
|  | MoCA | 24.99±2.80 | 25.52±2.62 | 1.89 | 0.175 | 0.013 |
| Attention |  |  |  |  |  |  |
|  | TMTA | 30.66±11.91 | 27.19±9.58 | 4.00 | *0.046* | 0.028 |
|  | ACS | 45.61±6.46 | 54.44±6.43 | 50.43 | *<0.001* | 0.262 |
| Executive function | |  |  |  |  |  |
|  | TMTB | 90.28±47.92 | 67.25±28.57 | 10.96 | *0.001* | 0.075 |
|  | FAB | 14.68±1.26 | 17.79±0.46 | 294.89 | *<0.001* | 0.682 |
| Memory |  |  |  |  |  |  |
|  | DST-f | 12.65±2.17 | 13.21±2.15 | 3.25 | 0.071 | 0.023 |
|  | DST-b | 10.33±2.84 | 11.15±2.65 | 2.62 | 0.098 | 0.019 |
|  | DST-s | 9.56±2.17 | 10.23±2.31 | 3.33 | 0.063 | 0.024 |
|  | DST | 32.53±6.07 | 34.60±5.73 | 4.80 | *0.026* | 0.035 |
| Anxiety |  |  |  |  |  |  |
|  | HAMA | 8.09±5.40 | 4.35±3.74 | 22.55 | *<0.001* | 0.136 |
| Depression |  |  |  |  |  |  |
|  | BDI | 4.41±4.34 | 3.46±4.13 | 2.02 | 0.182 | 0.013 |
| Affection |  |  |  |  |  |  |
|  | PANASp | 27.59±7.56 | 31.77±7.37 | 11.34 | *0.001* | 0.074 |
|  | PANASn | 21.36±7.40 | 16.75±5.34 | 16.53 | *<0.001* | 0.102 |
| Empathy |  |  |  |  |  |  |
|  | IRI | 49.29±9.52 | 55.87±10.51 | 15.19 | *<0.001* | 0.094 |

Group comparisons were performed using multivariate analysis of covariance (MANCOVA); *p* value < 0.05 was considered statistically significant. Italic type indicates statistically significant differences. ACS, Attentional Control Scale; BDI, Beck Depression Inventory; DST: Digit Span Test; DST-b, Wechsler Adult Intelligence Scale—Fourth Edition Digit Span-Backward; DST-f, Wechsler Adult Intelligence Scale—Fourth Edition Digit Span-Forward; DST-s, Wechsler Adult Intelligence Scale—Fourth Edition Digit Span-Sequencing; FAB, Frontal Assessment Battery; HAMA, Hamilton Anxiety Scale; HCs, healthy controls; IRI, Interpersonal Reactivity Index; MMSE, mini-mental state examination; MoCA, Montreal Cognitive Assessment; PANAS, Positive and Negative Affect Scale; PANASn, Positive and Negative Affect Scale-negative affect; PANASp, Positive and Negative Affect Scale-positive affect; TMTA, Trail-Making Test part A; TMTB, Trial-Making Test-part B.

**Table S3. Demographic, Clinical, Molecular Characteristics and neuropsychological Tests of Non-dominant vs. Dominant Hemisphere in Insular Subgroup Patients**

| **Variables** |  | **Non-dominant(n=14)** | **Dominant hemisphere(n=12)** | **t/Z** | ***p-*value** |
| --- | --- | --- | --- | --- | --- |
| **Demographic and clinical features** | |  |  |  |  |
|  | Gender(M) | 7(50.0%) | 6(50.0%) | - | 1.000 |
|  | Age | 39.86±8.39 | 42.58±10.46 | -0.738 | 0.468 |
|  | Education(y) | 13.50±3.50 | 13.42±2.78 | 0.066 | 0.948 |
|  | Smoking | 3(21.4%) | 3(25.0%) | - | 1.000 |
|  | Alcohol consumption | 3(21.4%) | 3(25.0%) | - | 1.000 |
|  | Hypertension | 1(7.1%) | 0 | - | 1.000 |
|  | Diabetes | 1(7.1%) | 0 | - | 1.000 |
|  | Operation history | 1(7.1%) | 3(25.0%) | - | 0.306 |
|  | History of other system disease | 4(28.6%) | 2(16.7%) | - | 0.652 |
|  | Disease course(mos) | 4.00±7.00 | 2.50±6.00 | -0.026 | 0.979 |
|  | WHO grade(high) | 2(14.3%) | 4(33.3%) | - | 0.365 |
|  | Epilepsy | 7(50.0%) | 6(50.0%) | - | 1.000 |
|  | ki-67(%) | 5.00±3.40 | 5.00±6.80 | -0.155 | 0.877 |
|  | 1p/19q co-deletion | 8(57.1%) | 4(33.3%) | - | 0.267 |
|  | MGMT methylation | 10(71.4%) | 7(58.3%) | - | 0.683 |
|  | TERT mutation | 8(57.1%) | 8(66.7%) | - | 0.701 |
|  | IDH mutation | 11(78.6%) | 7(58.3%) | - | 0.401 |
|  | Tumor size(cm3) | 60.81±67.04 | 66.35±44.73 | -0.243 | 0.810 |
| **Neuropsychological tests** | |  |  |  |  |
|  | MMSE | 29.00±2.00 | 28.00±1.00 | -1.378 | 0.168 |
|  | MoCA | 26.79±1.76 | 23.33±2.81 | 3.815 | *0.001* |
|  | TMTA | 26.86±7.87 | 28.08±8.07 | -0.391 | 0.699 |
|  | TMTB | 72.86±31.22 | 83.58±46.17 | -0.703 | 0.489 |
|  | DST-f | 13.07±2.09 | 12.58±2.02 | 0.602 | 0.553 |
|  | DST-b | 10.71±3.07 | 10.17±1.99 | 0.528 | 0.602 |
|  | DST-s | 10.36±2.21 | 9.17±1.53 | 1.572 | 0.129 |
|  | DST | 34.14±5.59 | 31.83±4.32 | 1.163 | 0.256 |
|  | FAB | 15.00±1.00 | 15.00±1.00 | -0.532 | 0.595 |
|  | HAMA | 7.29±4.71 | 7.83±5.57 | -0.272 | 0.788 |
|  | ACS | 44.36±6.87 | 46.92±7.05 | -0.936 | 0.359 |
|  | BDI | 2.00±5.00 | 2.50±5.00 | -0.052 | 0.959 |
|  | PANASp | 28.36±7.57 | 28.33±8.28 | 0.008 | 0.994 |
|  | PANASn | 19.71±8.13 | 22.67±7.76 | -0.943 | 0.355 |
|  | IRI | 49.07±7.95 | 52.75±5.69 | -1.335 | 0.194 |

“–”, not applicable; Italic type indicates statistically significant differences. ACS, Attentional Control Scale; BDI, Beck Depression Inventory; DST: Digit Span Test; DST-b, Wechsler Adult Intelligence Scale—Fourth Edition Digit Span-Backward; DST-f, Wechsler Adult Intelligence Scale—Fourth Edition Digit Span-Forward; DST-s, Wechsler Adult Intelligence Scale—Fourth Edition Digit Span-Sequencing; FAB, Frontal Assessment Battery; HAMA, Hamilton Anxiety Scale; HCs, healthy controls; IRI, Interpersonal Reactivity Index; MMSE, mini-mental state examination; MoCA, Montreal Cognitive Assessment; PANAS, Positive and Negative Affect Scale; PANASn, Positive and Negative Affect Scale-negative affect; PANASp, Positive and Negative Affect Scale-positive affect; TMTA, Trail-Making Test part A; TMTB, Trial-Making Test-part B.

**Table S4. Demographic, Clinical, Molecular Characteristics and neuropsychological Tests of IDH-wildtype vs. IDH-mutation in Insular Subgroup Patients**

| **Variables** |  | **IDH-wildtype(n=8)** | **IDH-mutation(n=18)** | **t/Z** | ***p*-value** |
| --- | --- | --- | --- | --- | --- |
| **Demographic and clinical features** | |  |  |  |  |
|  | Gender(M) | 5(62.5%) | 8(44.4%) | - | 0.673 |
|  | Age | 42.88±7.94 | 40.33±9.97 | 0.635 | 0.532 |
|  | Education(y) | 12.38±3.02 | 13.94±3.13 | -1.191 | 0.245 |
|  | Smoking | 2(25.0%) | 4(22.2%) | - | 1.000 |
|  | Alcohol consumption | 3(37.5%) | 3(16.7%) | - | 0.330 |
|  | Hypertension | 0 | 1(5.6%) | - | 1.000 |
|  | Diabetes | 0 | 1(5.6%) | - | 1.000 |
|  | Operation history | 0 | 4(22.2%) | - | 0.277 |
|  | History of other system disease | 1(12.5%) | 5(27.8%) | - | 0.628 |
|  | Disease course(mos) | 2.50±1.25 | 5.00±11.50 | -0.529 | 0.597 |
|  | WHO grade(high) | 3(37.5%) | 3(16.7%) | - | 0.330 |
|  | Epilepsy | 4(50.0%) | 9(50.0%) | - | 1.000 |
|  | ki-67(%) | 5.50±5.60 | 4.75±3.60 | -0.697 | 0.486 |
|  | 1p/19q co-deletion | 0 | 12(66.7%) | - | *0.002* |
|  | MGMT methylation | 3(37.5%) | 14(77.8%) | - | 0.078 |
|  | TERT mutation | 5(62.5%) | 11(61.1%) | - | 1.000 |
|  | Dominant hemisphere tumor | 5(62.5%) | 7(38.9%) | - | 0.401 |
|  | Tumor size(cm3) | 42.50±16.91 | 72.64±65.80 | -1.814 | 0.084 |
| **Neuropsychological tests** | |  |  |  |  |
|  | MMSE | 28.50±1.00 | 29.00±2.00 | -0.934 | 0.350 |
|  | MoCA | 25.13±2.23 | 25.22±3.15 | -0.078 | 0.938 |
|  | TMTA | 25.88±4.22 | 28.11±9.01 | -0.665 | 0.513 |
|  | TMTB | 58.50±40.00 | 76.00±40.00 | -0.917 | 0.359 |
|  | DST-f | 12.5±1.60 | 13.00±2.22 | -0.571 | 0.573 |
|  | DST-b | 10.00±1.00 | 10.72±3.08 | -1.113 | 0.279 |
|  | DST-s | 9.50±1.31 | 9.94±2.24 | -0.520 | 0.608 |
|  | DST | 31.88±1.25 | 33.61±6.03 | -1.167 | 0.257 |
|  | FAB | 14.50±1.00 |  | -1.580 | 0.114 |
|  | HAMA | 10.25±4.92 | 6.33±4.72 | 1.930 | 0.066 |
|  | ACS | 53.75±2.87 | 41.89±4.64 | 6.647 | *＜0.001* |
|  | BDI | 4.00±8.00 | 2.78±3.61 | 1.798 | 0.085 |
|  | PANASp | 21.38±4.07 | 31.44±6.96 | -3.790 | *0.001* |
|  | PANASn | 28.75±4.13 | 17.67±6.77 | 4.265 | *＜0.001* |
|  | IRI | 55.38±3.66 | 48.72±7.38 | 2.402 | *0.024* |

“–”, not applicable; Italic type indicates statistically significant differences. ACS, Attentional Control Scale; BDI, Beck Depression Inventory; DST: Digit Span Test; DST-b, Wechsler Adult Intelligence Scale—Fourth Edition Digit Span-Backward; DST-f, Wechsler Adult Intelligence Scale—Fourth Edition Digit Span-Forward; DST-s, Wechsler Adult Intelligence Scale—Fourth Edition Digit Span-Sequencing; FAB, Frontal Assessment Battery; HAMA, Hamilton Anxiety Scale; HCs, healthy controls; IRI, Interpersonal Reactivity Index; MMSE, mini-mental state examination; MoCA, Montreal Cognitive Assessment; PANAS, Positive and Negative Affect Scale; PANASn, Positive and Negative Affect Scale-negative affect; PANASp, Positive and Negative Affect Scale-positive affect; TMTA, Trail-Making Test part A; TMTB, Trial-Making Test-part B.

**Table S5. Demographic, Clinical, Molecular Characteristics and neuropsychological Tests of Non-epilepsy vs. Epilepsy in Frontal Subgroup Patients**

| **Variables** |  | **Non-epilepsy(n=33)** | **Epilepsy(n=19)** | **t/Z/χ2** | ***p*-value** |
| --- | --- | --- | --- | --- | --- |
| **Demographic and clinical features** | |  |  |  |  |
|  | Gender(M) | 17(51.5%) | 9(47.4%) | 0.083 | 0.773 |
|  | Age | 42.52±11.79 | 37.95±10.31 | 1.406 | 0.166 |
|  | Education(y) | 14.00±4.00 | 12.00±5.00 | -0.904 | 0.366 |
|  | Smoking | 5(15.2%) | 4(21.1%) | 0.026 | 0.872 |
|  | Alcohol consumption | 7(21.2%) | 8(42.1%) | 2.564 | 0.109 |
|  | Hypertension | 5(15.2%) | 0 | 1.680 | 0.195 |
|  | Diabetes | 2(6.1%) | 1(5.3%) | ＜0.001 | 1.000 |
|  | Operation history | 11(33.3%) | 5(26.3) | 0.279 | 0.598 |
|  | History of other system disease | 15(45.5%) | 8(42.1%) | 0.055 | 0.815 |
|  | Disease course(mos) | 2.00±6.50 | 1.00±3.92 | -1.577 | 0.115 |
|  | Dominant hemisphere tumor | 15(45.5%) | 12(63.2%) | 1.514 | 0.219 |
|  | WHO grade(high) | 13(39.4%) | 7(36.8%) | 0.033 | 0.855 |
|  | ki-67(%) | 5.00±22.5 | 7.50±19.00 | -0.349 | 0.727 |
|  | 1p/19q co-deletion | 10(30.3%) | 9(47.4%) | 1.514 | 0.218 |
|  | MGMT methylation | 24(72.7%) | 9(47.4%) | 3.344 | 0.067 |
|  | TERT mutation | 15(45.5%) | 12(63.2%) | 1.514 | 0.219 |
|  | IDH mutation | 22(66.7%) | 14(73.7%) | 0.279 | 0.598 |
|  | Tumor size(cm3) | 36.83±58.89 | 63.37±61.19 | -0.969 | 0.332 |
| **Neuropsychological tests** | |  |  |  |  |
|  | MMSE | 29.00±2.00 | 29.00±4.00 | -0.236 | 0.814 |
|  | MoCA | 25.58±2.33 | 23.47±3.32 | 2.434 | *0.021* |
|  | TMTA | 31.30±14.13 | 32.58±10.76 | -0.340 | 0.735 |
|  | TMTB | 88.67±51.40 | 98.63±44.66 | -0.705 | 0.484 |
|  | DST-f | 12.97±2.08 | 12.32±2.83 | 0.954 | 0.345 |
|  | DST-b | 10.94±3.15 | 9.79±2.80 | 1.318 | 0.194 |
|  | DST-s | 10.15±2.15 | 8.42±2.22 | 2.761 | *0.008* |
|  | DST | 34.06±6.19 | 30.53±7.14 | 1.873 | 0.067 |
|  | FAB | 15.06±1.25 | 14.05±1.96 | 2.270 | *0.028* |
|  | HAMA | 7.58±5.14 | 8.72±6.15 | -0.710 | 0.481 |
|  | ACS | 46.21±7.66 | 46.44±3.55 | -0.148 | 0.883 |
|  | BDI | 3.00±5.00 | 3.50±8.00 | -0.258 | 0.796 |
|  | PANASp | 29.38±7.85 | 23.28±7.32 | 2.700 | *0.010* |
|  | PANASn | 20.69±6.72 | 23.17±9.83 | -0.952 | 0.350 |
|  | IRI | 47.12±10.07 | 49.61±11.83 | -0.793 | 0.432 |

Italic type indicates statistically significant differences. ACS, Attentional Control Scale; BDI, Beck Depression Inventory; DST: Digit Span Test; DST-b, Wechsler Adult Intelligence Scale—Fourth Edition Digit Span-Backward; DST-f, Wechsler Adult Intelligence Scale—Fourth Edition Digit Span-Forward; DST-s, Wechsler Adult Intelligence Scale—Fourth Edition Digit Span-Sequencing; FAB, Frontal Assessment Battery; HAMA, Hamilton Anxiety Scale; HCs, healthy controls; IRI, Interpersonal Reactivity Index; MMSE, mini-mental state examination; MoCA, Montreal Cognitive Assessment; PANAS, Positive and Negative Affect Scale; PANASn, Positive and Negative Affect Scale-negative affect; PANASp, Positive and Negative Affect Scale-positive affect; TMTA, Trail-Making Test part A; TMTB, Trial-Making Test-part B.

**Table S6. Demographic, Clinical, Molecular Characteristics and neuropsychological Tests of Low Grade vs. High Grade in Frontal Subgroup Patients**

| **Variables** |  | **Low grade(n=32)** | **High grade(n=20)** | **t/Z/χ2** | ***p*-value** |
| --- | --- | --- | --- | --- | --- |
| **Demographic and clinical features** | | |  |  |  |
|  | Gender(M) | 13(40.6%) | 13(65.0%) | 2.925 | 0.087 |
|  | Age | 40.72±10.95 | 41.05±12.34 | -0.101 | 0.920 |
|  | Education(y) | 13.22±3.08 | 13.40±2.82 | 0.213 | 0.832 |
|  | Smoking | 5(15.6%) | 4(20.0%) | 0.001 | 0.977 |
|  | Alcohol consumption | 8(25.0%) | 7(35.0%) | 0.600 | 0.439 |
|  | Hypertension | 4(12.5%) | 1(5.0%) | 0.167 | 0.682 |
|  | Diabetes | 1(3.1%) | 2(10.0%) | 0.179 | 0.672 |
|  | Operation history | 11(34.4%) | 5(25.0%) | 0.508 | 0.476 |
|  | History of other system disease | 15(46.9%) | 8(40.0%) | 0.236 | 0.627 |
|  | Disease course(mos) | 1.50±11.30 | 1.25±1.33 | -1.418 | 0.156 |
|  | Dominant hemisphere tumor | 14(43.8%) | 13(65.0%) | 2.226 | 0.136 |
|  | Epilepsy | 12(37.5%) | 7(35.0%) | 0.033 | 0.855 |
|  | ki-67(%) | 4.37±3.03 | 32.16±19.76 | -6.242 | *＜0.001* |
|  | 1p/19q co-deletion | 14(43.8%) | 5(25.0%) | 1.866 | 0.172 |
|  | MGMT methylation | 21(65.6%) | 12(60.0%) | 0.168 | 0.682 |
|  | TERT mutation | 14(43.8%) | 13(65.0%) | 2.226 | 0.136 |
|  | IDH mutation | 25(78.1%) | 11(55.0%) | 3.090 | 0.079 |
|  | Tumor size(cm3) | 21.42±56.32 | 71.67±58.93 | -3.085 | *0.002* |
| **Neuropsychological tests** | |  |  |  |  |
|  | MMSE | 29.00±2.00 | 29.00±3.00 | -0.408 | 0.683 |
|  | MoCA | 24.53±2.69 | 25.25±3.21 | -0.870 | 0.388 |
|  | TMTA | 28.50±15.00 | 28.00±20.00 | -0.527 | 0.598 |
|  | TMTB | 83.50±87.00 | 74.50±55.00 | -0.771 | 0.441 |
|  | DST-f | 12.56±2.21 | 13.00±2.66 | -0.642 | 0.524 |
|  | DST-b | 10.19±3.30 | 11.05±2.61 | -0.991 | 0.326 |
|  | DST-s | 9.41±2.31 | 9.70±2.36 | -0.442 | 0.660 |
|  | DST | 32.16±6.63 | 33.75±6.89 | -0.831 | 0.410 |
|  | FAB | 15.00±1.00 | 15.00±1.00 | -0.180 | 0.857 |
|  | HAMA | 8.66±6.03 | 6.84±4.32 | 1.146 | 0.257 |
|  | ACS | 46.91±7.42 | 45.26±4.42 | 0.875 | 0.386 |
|  | BDI | 4.78±4.65 | 3.89±3.40 | 0.723 | 0.473 |
|  | PANASp | 27.03±8.73 | 27.42±7.31 | -0.162 | 0.872 |
|  | PANASn | 22.10±8.54 | 20.74±7.08 | 0.582 | 0.563 |
|  | IRI | 52.09±9.76 | 41.11±8.52 | 4.069 | *＜0.001* |

Italic type indicates statistically significant differences. ACS, Attentional Control Scale; BDI, Beck Depression Inventory; DST: Digit Span Test; DST-b, Wechsler Adult Intelligence Scale—Fourth Edition Digit Span-Backward; DST-f, Wechsler Adult Intelligence Scale—Fourth Edition Digit Span-Forward; DST-s, Wechsler Adult Intelligence Scale—Fourth Edition Digit Span-Sequencing; FAB, Frontal Assessment Battery; HAMA, Hamilton Anxiety Scale; HCs, healthy controls; IRI, Interpersonal Reactivity Index; MMSE, mini-mental state examination; MoCA, Montreal Cognitive Assessment; PANAS, Positive and Negative Affect Scale; PANASn, Positive and Negative Affect Scale-negative affect; PANASp, Positive and Negative Affect Scale-positive affect; TMTA, Trail-Making Test part A; TMTB, Trial-Making Test-part B.

**Table S7. Demographic, Clinical, Molecular Characteristics and neuropsychological Tests of IDH-wildtype vs. IDH-mutation in Frontal Subgroup Patients**

| **Variables** |  | **IDH-wildtype(n=16)** | **IDH-mutation(n=36)** | **t/Z/χ2** | ***p*-value** |
| --- | --- | --- | --- | --- | --- |
| **Demographic and clinical features** | |  |  |  |  |
|  | Gender(M) | 7(43.8%) | 19(52.8%) | 0.361 | 0.548 |
|  | Age | 42.88±12.80 | 39.94±10.77 | 0.854 | 0.397 |
|  | Education(y) | 12.25±3.19 | 13.75±2.76 | -1.723 | 0.091 |
|  | Smoking | 2(12.5%) | 7(19.4%) | 0.046 | 0.831 |
|  | Alcohol consumption | 4(25.0%) | 11(30.6%) | 0.006 | 0.939 |
|  | Hypertension | 1(6.3%) | 4(11.1%) | 0.002 | 0.969 |
|  | Diabetes | 1(6.3%) | 2(5.6%) | ＜0.001 | 1.000 |
|  | Operation history | 3(18.8%) | 13(36.1%) | 0.858 | 0.354 |
|  | History of other system disease | 4(25.0%) | 19(52.8%) | 3.465 | 0.063 |
|  | Disease course(mos) | 2.00±3.88 | 1.00±7.05 | -0.419 | 0.675 |
|  | WHO grade(high) | 9(56.3%) | 11(30.6%) | 3.09 | 0.079 |
|  | Epilepsy | 5(31.3%) | 14(38.9%) | 0.279 | 0.598 |
|  | ki-67(%) | 23.50±37.10 | 5.00±8.50 | -0.705 | 0.481 |
|  | 1p/19q co-deletion | 2(12.5%) | 17(47.2%) | 5.759 | *0.016* |
|  | MGMT methylation | 5(31.3%) | 28(77.8%) | 10.341 | *0.001* |
|  | TERT mutation | 6(60.0%) | 17(58.6%) | ＜0.001 | 1.000 |
|  | Dominant hemisphere tumor | 8(50.0%) | 19(52.8%) | 0.034 | 0.853 |
|  | Tumor size(cm3) | 30.39±57.72 | 46.49±65.77 | -1.735 | 0.083 |
| **Neuropsychological tests** | |  |  |  |  |
|  | MMSE | 29.00±3.00 | 29.00±2.00 | -0.102 | 0.918 |
|  | MoCA | 24.06±2.54 | 25.14±3.01 | -1.246 | 0.219 |
|  | TMTA | 38.94±13.89 | 28.58±11.22 | 2.852 | *0.006* |
|  | TMTB | 108.31±54.21 | 85.19±45.25 | 1.599 | 0.116 |
|  | DST-f | 12.19±2.01 | 12.97±2.51 | -1.101 | 0.276 |
|  | DST-b | 10.19±2.74 | 10.67±3.21 | -0.519 | 0.606 |
|  | DST-s | 9.25±2.72 | 9.64±2.14 | -0.556 | 0.581 |
|  | DST | 31.63±6.57 | 33.28±6.80 | -0.817 | 0.418 |
|  | FAB | 15.06±2.32 | 14.53±1.16 | 0.874 | 0.394 |
|  | HAMA | 7.88±5.11 | 8.06±5.64 | -0.110 | 0.913 |
|  | ACS | 48.00±6.88 | 45.53±6.13 | 1.293 | 0.202 |
|  | BDI | 3.50±8.00 | 3.00±6.00 | -0.399 | 0.690 |
|  | PANASp | 25.94±7.19 | 27.64±8.40 | -0.703 | 0.485 |
|  | PANASn | 23.50±8.00 | 20.75±7.70 | 1.174 | 0.246 |
|  | IRI | 46.81±10.23 | 48.31±10.91 | -0.464 | 0.645 |

Italic type indicates statistically significant differences. ACS, Attentional Control Scale; BDI, Beck Depression Inventory; DST: Digit Span Test; DST-b, Wechsler Adult Intelligence Scale—Fourth Edition Digit Span-Backward; DST-f, Wechsler Adult Intelligence Scale—Fourth Edition Digit Span-Forward; DST-s, Wechsler Adult Intelligence Scale—Fourth Edition Digit Span-Sequencing; FAB, Frontal Assessment Battery; HAMA, Hamilton Anxiety Scale; HCs, healthy controls; IRI, Interpersonal Reactivity Index; MMSE, mini-mental state examination; MoCA, Montreal Cognitive Assessment; PANAS, Positive and Negative Affect Scale; PANASn, Positive and Negative Affect Scale-negative affect; PANASp, Positive and Negative Affect Scale-positive affect; TMTA, Trail-Making Test part A; TMTB, Trial-Making Test-part B.

**Table S8. Demographic, Clinical, Molecular Characteristics and neuropsychological Tests of No Co-deletion vs. 1p/19q Co-deletion in Frontal Subgroup Patients**

| **Variables** |  | | **No co-deletion(n=33)** | **1p/19q co-deletion(n=19)** | **t/Z/χ2** | ***p*-value** |
| --- | --- | --- | --- | --- | --- | --- |
| **Demographic and clinical features** | | |  |  |  |  |
|  | | Gender(M) | 16(48.5%) | 10(52.6%) | 0.083 | 0.773 |
|  | | Age | 43.03±11.82 | 37.05±9.75 | 1.867 | 0.068 |
|  | | Education(y) | 13.15±2.96 | 13.53±3.01 | -0.437 | 0.664 |
|  | | Smoking | 7(21.2%) | 2(10.5%) | 0.36 | 0.548 |
|  | | Alcohol consumption | 11(33.3%) | 4(21.1%) | 0.886 | 0.347 |
|  | | Hypertension | 4(12.1%) | 1(5.3%) | 0.102 | 0.749 |
|  | | Diabetes | 2(6.1%) | 1(5.3%) | ＜0.001 | 1.000 |
|  | | Operation history | 11(33.3%) | 5(26.3%) | 0.279 | 0.598 |
|  | | History of other system disease | 15(45.5%) | 8(42.1%) | 0.055 | 0.815 |
|  | | Disease course(mos) | 2.00±6.50 | 1.00±1.50 | -1.916 | 0.055 |
|  | | WHO grade(high) | 15(45.5%) | 5(26.3%) | 1.866 | 0.172 |
|  | | Epilepsy | 10(30.3%) | 9(47.4%) | 1.514 | 0.218 |
|  | | ki-67(%) | 5.00±30.00 | 7.50±9.50 | -0.419 | 0.675 |
|  | | IDH mutation | 19(57.6%) | 17(89.5%) | 5.759 | *0.016* |
|  | | MGMT methylation | 18(54.5%) | 15(78.9%) | 3.096 | 0.078 |
|  | | TERT mutation | 12(36.4%) | 15(78.9%) | 8.759 | *0.003* |
|  | | Dominant hemisphere tumor | 18(54.5%) | 9(47.4%) | 0.249 | 0.618 |
|  | | Tumor size(cm3) | 26.49±65.09 | 50.19±47.87 | -1.017 | 0.309 |
| **Neuropsychological tests** | | |  |  |  |  |
|  | | MMSE | 29.00±3.00 | 29.00±2.00 | -0.785 | 0.432 |
|  | | MoCA | 24.58±2.75 | 25.21±3.16 | -0.759 | 0.451 |
|  | | TMTA | 35.18±13.07 | 25.84±10.45 | 2.659 | *0.010* |
|  | | TMTB | 102.27±53.76 | 75.00±33.52 | 1.995 | 0.052 |
|  | | DST-f | 11.94±2.36 | 14.11±1.73 | -3.494 | *0.001* |
|  | | DST-b | 9.48±2.77 | 12.32±2.71 | -3.574 | *0.001* |
|  | | DST-s | 9.06±2.11 | 10.32±2.50 | -1.934 | 0.059 |
|  | | DST | 30.48±6.10 | 36.74±5.94 | -3.592 | *0.001* |
|  | | FAB | 15.00±1.00 | 15.00±1.00 | -0.252 | 0.801 |
|  | | HAMA | 8.21±5.09 | 7.63±6.11 | 0.368 | 0.714 |
|  | | ACS | 45.97±7.02 | 46.84±5.31 | -0.469 | 0.641 |
|  | | BDI | 4.36±4.62 | 4.58±3.36 | -0.178 | 0.860 |
|  | | PANASp | 28.18±8.41 | 25.26±7.13 | 1.272 | 0.209 |
|  | | PANASn | 22.24±8.08 | 20.47±7.43 | 0.782 | 0.438 |
|  | | IRI | 48.15±9.83 | 47.32±12.16 | 0.271 | 0.788 |

Boldface type indicates statistically significant differences. ACS, Attentional Control Scale; BDI, Beck Depression Inventory; DST: Digit Span Test; DST-b, Wechsler Adult Intelligence Scale—Fourth Edition Digit Span-Backward; DST-f, Wechsler Adult Intelligence Scale—Fourth Edition Digit Span-Forward; DST-s, Wechsler Adult Intelligence Scale—Fourth Edition Digit Span-Sequencing; FAB, Frontal Assessment Battery; HAMA, Hamilton Anxiety Scale; HCs, healthy controls; IRI, Interpersonal Reactivity Index; MMSE, mini-mental state examination; MoCA, Montreal Cognitive Assessment; PANAS, Positive and Negative Affect Scale; PANASn, Positive and Negative Affect Scale-negative affect; PANASp, Positive and Negative Affect Scale-positive affect; TMTA, Trail-Making Test part A; TMTB, Trial-Making Test-part B

**Table S9. Demographic, Clinical, Molecular Characteristics and neuropsychological Tests of TERT-wildtype vs. TERT-mutation in Frontal Subgroup Patients**

| **Variables** |  | **TERT wildtype(n=25)** | **TERT mutation(n=27)** | **t/Z/χ2** | **p-value** |
| --- | --- | --- | --- | --- | --- |
| **Demographic and clinical features** | |  |  |  |  |
|  | Gender(M) | 10(40.0%) | 16(59.3%) | 1.926 | 0.165 |
|  | Age | 38.48±10.74 | 43.04±11.72 | -1.458 | 0.151 |
|  | Education(y) | 13.84±2.94 | 12.78±2.93 | 1.305 | 0.198 |
|  | Smoking | 3(12.0%) | 6(22.2%) | 0.368 | 0.544 |
|  | Alcohol consumption | 6(24.0%) | 9(33.3%) | 0.551 | 0.458 |
|  | Hypertension | 1(4.0%) | 4(14.8%) | 0.724 | 0.395 |
|  | Diabetes | 0 | 3(11.1%) | 1.258 | 0.262 |
|  | Operation history | 8(32.0%) | 8(29.6%) | 0.034 | 0.853 |
|  | History of other system disease | 9(36.0%) | 14(51.9%) | 1.322 | 0.25 |
|  | Disease course(mos) | 2.00±14.42 | 1.00±2.00 | -0.646 | 0.519 |
|  | WHO grade(high) | 7(28.0%) | 13(48.1%) | 2.226 | 0.136 |
|  | Epilepsy | 7(28.0%) | 12(44.4%) | 1.514 | 0.219 |
|  | ki-67(%) | 5.00±29.80 | 11.00±19.40 | -1.558 | 0.119 |
|  | IDH mutation | 16(64.0%) | 20(74.1%) | 0.618 | 0.432 |
|  | MGMT methylation | 13(52.0%) | 20(74.1%) | 2.728 | 0.099 |
|  | 1p/19q co-deletion | 4(16.0%) | 15(55.6%) | 8.759 | *0.003* |
|  | Dominant hemisphere tumor | 13(52.0%) | 14(51.9%) | ＜0.001 | 1.000 |
|  | Tumor size(cm3) | 25.44±85.22 | 57.61±39.67 | -1.156 | 0.247 |
| **Neuropsychological tests** | |  |  |  |  |
|  | MMSE | 29.00±2.00 | 28.00±3.00 | -1.287 | 0.198 |
|  | MoCA | 25.20±2.45 | 24.44±3.25 | 0.941 | 0.351 |
|  | TMTA | 29.72±10.44 | 33.67±14.77 | -1.105 | 0.275 |
|  | TMTB | 75.00±62.00 | 95.00±61.00 | -1.456 | 0.145 |
|  | DST-f | 12.92±2.24 | 12.56±2.53 | 0.549 | 0.586 |
|  | DST-b | 10.44±3.22 | 10.59±2.95 | -0.178 | 0.859 |
|  | DST-s | 9.60±2.29 | 9.44±2.38 | 0.24 | 0.811 |
|  | DST | 32.96±6.72 | 32.59±6.83 | 0.195 | 0.846 |
|  | FAB | 15.00±2.00 | 15.00±1.00 | -0.845 | 0.398 |
|  | HAMA | 8.48±5.77 | 7.56±5.16 | 0.61 | 0.545 |
|  | ACS | 46.72±8.19 | 45.89±4.26 | 0.464 | 0.645 |
|  | BDI | 3.00±8.00 | 3.00±6.00 | -0.018 | 0.985 |
|  | PANASp | 29.20±8.33 | 25.19±7.35 | 1.847 | 0.071 |
|  | PANASn | 22.12±8.44 | 21.11±7.33 | 0.461 | 0.647 |
|  | IRI | 53.48±8.40 | 42.63±9.89 | 4.248 | *＜0.001* |

Italic type indicates statistically significant differences. ACS, Attentional Control Scale; BDI, Beck Depression Inventory; DST: Digit Span Test; DST-b, Wechsler Adult Intelligence Scale—Fourth Edition Digit Span-Backward; DST-f, Wechsler Adult Intelligence Scale—Fourth Edition Digit Span-Forward; DST-s, Wechsler Adult Intelligence Scale—Fourth Edition Digit Span-Sequencing; FAB, Frontal Assessment Battery; HAMA, Hamilton Anxiety Scale; HCs, healthy controls; IRI, Interpersonal Reactivity Index; MMSE, mini-mental state examination; MoCA, Montreal Cognitive Assessment; PANAS, Positive and Negative Affect Scale; PANASn, Positive and Negative Affect Scale-negative affect; PANASp, Positive and Negative Affect Scale-positive affect; TMTA, Trail-Making Test part A; TMTB, Trial-Making Test-part B
